# Mediterranean Diet and Micronutrient Adequacy in Relation to Relapsing‐Remitting Multiple Sclerosis Risk: A Case‐Control Study

**DOI:** 10.1002/hsr2.73139

**Published:** 2026-08-30

**Authors:** Maryam Ahmadi‐Khorram, Alireza Hatami, Mostafa Shahraki Jazinaki, Yasaman Aali, Maedeh Nojoumi, Fatemeh Keykhaei, Ali Jafarzadeh Esfehani, Mohsen Nematy

**Affiliations:** ^1^ Department of Clinical Nutrition, School of Nutritional Sciences and Dietetics Tehran University of Medical Sciences Tehran Iran; ^2^ Students' Scientific Research Center (SSRC) Tehran University of Medical Sciences Tehran Iran; ^3^ Department of Clinical Nutrition, School of Nutrition and Food Science, Food Security Research Center Isfahan University of Medical Sciences Isfahan Iran; ^4^ Students' Research Committee Isfahan University of Medical Sciences Isfahan Iran; ^5^ Department of Nutrition, Faculty of Medicine Mashhad University of Medical Sciences Mashhad Iran; ^6^ Student Research Committee Mashhad University of Medical Sciences Mashhad Iran; ^7^ Metabolic Syndrome Research Center Mashhad University of Medical Sciences Mashhad Iran

**Keywords:** food frequency questionnaire, Mediterranean diet, micronutrient adequacy, multiple sclerosis, nutritional epidemiology

## Abstract

**Background:**

Multiple sclerosis (MS) is a chronic autoimmune and neurodegenerative disease influenced by environmental factors, including diet. The Mediterranean Diet (MeDi), rich in anti‐inflammatory and antioxidant compounds, and micronutrient adequacy may reduce MS risk and progression.

**Aim:**

This study aims to examine the associations between the Mediterranean Diet Score (MDS), Mean Adequacy Ratio (MAR), and MS risk in a case‐control setting.

**Methods:**

This case‐control study included 197 newly diagnosed relapsing‐remitting MS patients (aged 18–65 years) from Iran, and 200 matched controls without neurological diseases. Dietary intake was assessed using a validated semi‐quantitative food frequency questionnaire. MDS was calculated based on nine dietary components, and MAR was computed as the average Nutrient Adequacy Ratio for seven micronutrients. Adjusted binary logistic regression estimated odds ratios (ORs) for MS risk across MDS and MAR tertiles.

**Results:**

Higher MDS and MAR were associated with significantly reduced MS risk. Individuals in the highest MDS tertile had 47% lower odds of MS (OR: 0.53, 95% CI: 0.29–0.98, *p* = 0.04), and those in the highest MAR tertile had 75% lower odds (OR: 0.25, 95% CI: 0.10–0.62, *p* = 0.002) compared to the lowest tertiles after adjustments. No significant differences in total energy or macronutrient intake were observed between groups.

**Conclusion:**

Higher adherence to the Mediterranean Diet and better micronutrient adequacy are associated with a reduced risk of MS. These findings highlight the potential of dietary interventions as a cost‐effective strategy for MS prevention, warranting further longitudinal and interventional studies to establish causality and inform public health strategies.

**Trial Registration:** The Mashhad University of Medical Sciences approved the study with numbers IR.MUMS.REC.1393.182.

## Summary

- High Mediterranean Diet Score linked to 47% lower MS risk.
- High Mean Adequacy Ratio tied to 75% reduced MS odds.
- Findings support dietary interventions for MS prevention.

## 1 Introduction

Multiple sclerosis (MS) is a chronic autoimmune and neurodegenerative disease that affects the central nervous system, causing symptoms such as fatigue, motor impairment, and cognitive decline. With a global prevalence of approximately 35.9 per 100,000 and impacting around 2.8 million individuals [1], MS poses significant challenges to patients' quality of life and places considerable economic and social burdens on society [2]. While genetic factors play a crucial role in MS susceptibility, environmental influences, including diet, have been increasingly recognized as modifiable risk factors that can affect disease onset and progression [3].

Among various dietary patterns, the Mediterranean Diet (MeDi) has garnered attention for its potential neuroprotective effects. Characterized by high consumption of fruits, vegetables, whole grains, legumes, fish, and olive oil, the MeDi is rich in anti‐inflammatory and antioxidant compounds that may mitigate the inflammatory processes underlying MS [4]. The Mediterranean Diet Score (MDS) is a tool used to quantify adherence to this dietary pattern, with higher scores linked to reduced risks of chronic diseases, including neurodegenerative disorders [5]. Furthermore, the MeDi provides essential micronutrients, including vitamin D, vitamin B12, selenium, zinc, magnesium, vitamin C, and vitamin B9, which are crucial for immune function and neuroprotection. The Micronutrient Adequacy Ratio (MAR) assesses the adequacy of these nutrient intakes, and studies have suggested that adequate micronutrient levels may be associated with reduced inflammation and less severe MS outcomes [6, 7, 8].

Recent research has further supported the association between the MeDi and MS. For instance, Felicetti et al. found that higher adherence to the MeDi was associated with lower disability scores in people with MS [9]. Additionally, a recent study indicated that the MeDi might play a protective role against the development of MS [10]. These findings underscore the importance of dietary factors in MS management and highlight the need for further investigation into how specific dietary patterns and micronutrient intakes influence MS risk and progression.

This case‐control study aims to examine whether higher MDS and MAR are associated with a reduced risk of MS in newly diagnosed patients compared to healthy controls. By elucidating the relationship between diet, micronutrient adequacy, and MS, this research could provide valuable insights into dietary strategies that may help in the prevention and management of MS.

## 2 Materials and Methods

Investigating the associations between adherence to MD and MAR and the risk of MS was the main objective of the present research. All enrolled participants agreed to the inclusion of this research and signed the informed consent form.

### 2.1 Study Design

This case‐control study involves 197 new diagnostic relapsing–remitting MS (RRMS) patients aged 18–65 years old with no changes in their diets in the past 6 months.

The patients were selected from the MS Association Registry in 2015.

A total of 200 potential controls were included to our study based on following inclusion criteria: having no acute medical conditions or neurological diseases, be same in hospitalized center with cases. The exclusion criteria of the control group included intentional dietary modifications, pregnancy, and lactation. Also, the exclusion criteria for both groups were starting adherence to a specific dietary approach within the last 12 months, consuming any dietary supplements, and energy intake of less than 800 or higher than 4200 kcal per day. Matching of the control group with cases was performed based on sex, body mass index (BMI), education levels, and age (in 10‐year groups).

### 2.2 Data Collection

#### 2.2.1 Demographic and Body Composition Assessments

Smoking habits and demographic data were collected by interviewing the participants.

Weight and body composition were assessed using a body impedance analyzer (Tanita BC‐418) for each included individual when they were without shoes and wearing minimal clothes.

In addition, anthropometric measurements were performed according to the CDC anthropometry procedures manual 2007 of the National Health and Nutrition Examination Survey (NHANES). Height measurement was performed for participants in Frankfurt Plane using the stadiometer with an accuracy of 0.5 cm. Furthermore, the midpoint of the iliac crest and the lowest limb were measured twice to obtain the waist circumference.

#### 2.2.2 Dietary Intake Assessments

Usual food intakes were recorded by face‐to‐face personal interview using a semi‐quantitative food frequency questionnaire (FFQ) and were described in our previous study [11]. Also, this FFQ was valid in Iranian populations, as was reported in Ghazizahedi et al. [12]. The frequency of each food item intake was converted to daily intake. Also, by using household measures, the serving size of each recorded food was converted to grams. Nutritionist IV software package (First Databank Inc., Hearst Corp.) was used to estimate nutrient intakes from foods.

### 2.3 Mediterranean Diet Score (MDS) Calculation

The MDS was calculated according to the Trichopoulou et al. [13, 14]. approach by using nine dietary components. In this method, MDS was calculated based on six positive components, including nuts, ratio of monounsaturated fatty acids (MUFAs) to saturated fatty acids (SFAs), legumes, fruit, vegetable, and fish, and three negative components, including dairy, grains, and meats, including red meat, processed meat, and poultry meat. Participants received one score if they had a higher intake of median for positive components or a lower intake of median for negative components. In addition, if they had a higher dietary intake of negative or had a lower intake of positive components, they received a zero score. Finally, by summing the scores of all components, the MDS was calculated, which ranges from 0 to 9.

### 2.4 Mean Adequacy Ratio Calculation

The Mean Adequacy Ratio (MAR) was calculated to assess the diet quality [15] by using the Nutrient Adequacy Ratio (NAR) of 7 nutrients, including magnesium, zinc, vitamin B12, vitamin C, selenium, vitamin D, and folate [16, 17]. NAR was calculated by dividing the Daily nutrient intake by the Reference intake for each nutrient. To avoid compensating for a nutrient with an NAR exceeding 1 with a nutrient having a lower NAR, the NAR values are truncated at 1. Finally, the average of all‐NAR values was used to calculate MAR based on the following formula: MAR = ΣNAR (each truncated at 1)/number of nutrients [18].

### 2.5 Statistical Analysis

All analyses were performed using SPSS version 18. Also, *p* value < 0.05 was interpreted as statistical significance in all conducted analyses. In the present study, categorical and continuous variables were presented as frequency (%), and mean ± standard deviation (SD), respectively. Comparisons between categorical variables between the case and control group, as well as across the MAR and MD tertiles, were performed using the *χ* ^2^ test. Also, continuous variables with normal distribution were compared between cases and control groups as well across the MAR and MD tertiles by conducting the independent sample *t*‐test and one‐way ANOVA test, respectively. However, comparing continuous variables without normal distribution was performed by the Mann–Whitney *U* test for comparing case and control, and the Kruskal–Wallis test for comparing the tertiles of MAR and MD with each other. Binary logistic regression was performed to investigate the association between adherence to the MD or MAR and risk of MS by reporting the odds ratio (OR) with 95% confidence interval (CI). In addition, multivariable analysis was conducted to assess these relationships, followed by adjusting for the cofounders, including age, sex, smoking, and energy intake. In all crude and adjusted models, the first tertiles of MAR and MD were considered as reference. Due to the use of secondary data in this study, the power of the analyses for investigating the association between the highest adherence to the MAR and MD and the risk of MS, compared to the first tertile, was calculated using the GPower 3.1 software. In the fully adjusted models with the most covariates, the statistical powers were 100% and 99%, respectively, which are considered acceptable according to the cutoff points proposed by Cohen [19].

## 3 Results

**Figure 1:**
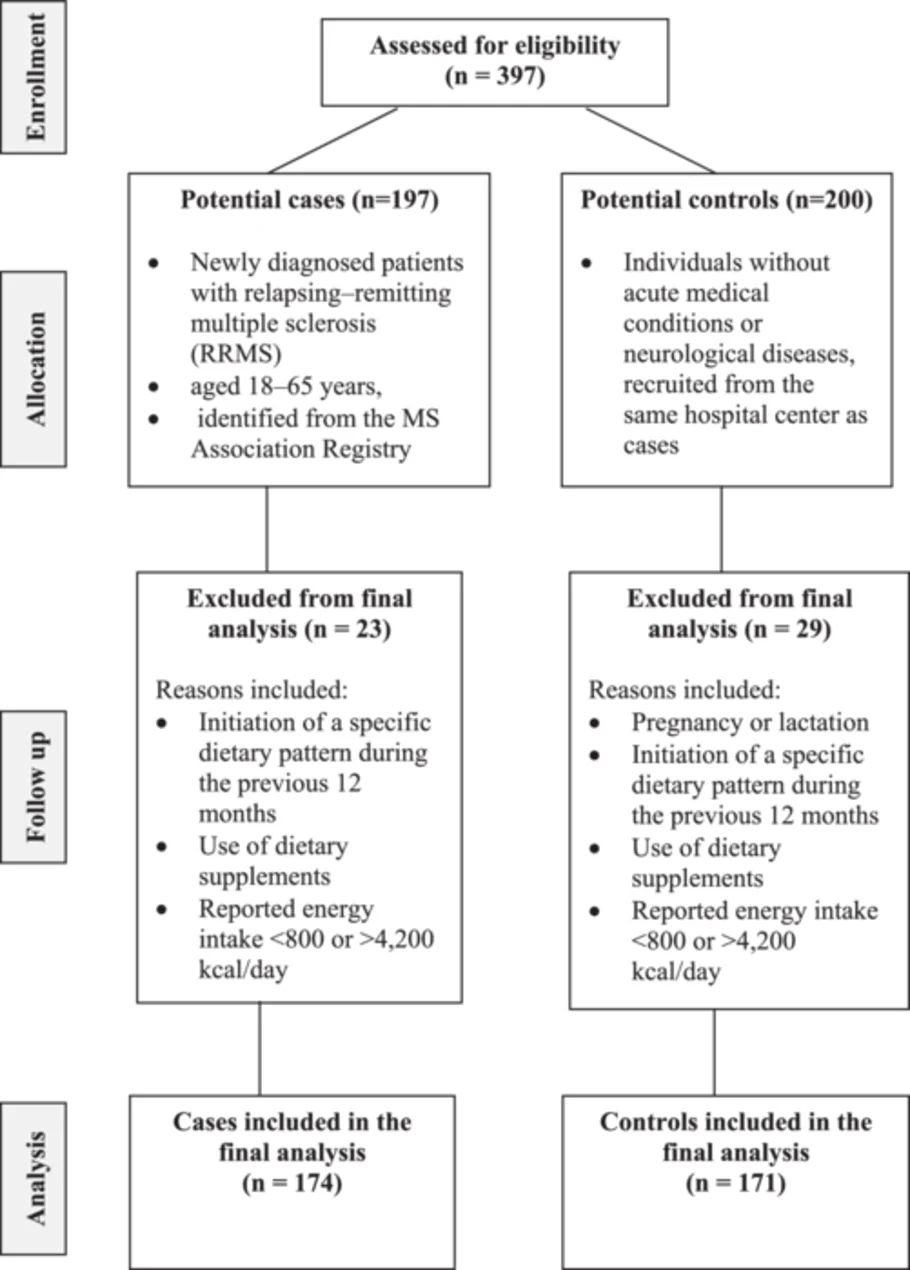
Flow diagram of participant selection. A total of 397 participants were initially assessed, including 197 newly diagnosed patients with relapsing–remitting multiple sclerosis (RRMS) and 200 control participants without neurological disease. In the final analysis, 174 cases and 171 controls were included.

Table 1 summarizes the characteristics of the participants in the case and control groups. Compared to the control groups, FM and the prevalence of smoking were significantly higher in cases. However, FFM was significantly higher in control groups than in individuals with MS. No significant differences were observed in other variables between these groups, including age, gender, BMI, and WC. Also, as shown in Table 2, there were no significant differences in total energy intake as well as macronutrient consumption, including carbohydrate, fat, protein, and fiber (Figure 1).

**Table 1:** Characteristics of participants across case and control groups.

Variables	Case group (*N* = 174)	Control group (*N* = 171)	*p* value
Continuous variables	Mean ± SD	Mean ± SD	
Age (year)	32.87 ± 8.85	32.00 ± 8.46	0.29
Weight (kg)	65.68 ± 14.54	66.21 ± 14.38	0.55
Height (cm)	163.14 ± 8.77	161.57 ± 8.36	0.10
BMI (kg/m^2^)	24.83 ± 5.57	25.33 ± 5.08	0.24
WC (cm)	83.40 ± 12.69	82.71 ± 12.93	0.55
Fat (%)	28.80 ± 7.85	26.70 ± 8.89	**0.03**
FFM	42.45 ± 7.96	47.50 ± 9.24	**< 0.001**
MAR	1.12 ± 0.11	1.13 ± 0.10	0.89
MD	4.37 ± 1.61	4.66 ± 1.53	0.15

Categorical variables	*N* (%)	*N* (%)	*p* value
Gender			
Female	135 (49.3)	139 (50.7)	0.39
Male	39 (54.9)	32 (45.1)	
Smoking			
Yes	40 (71.4)	16 (28.6)	**0.001**
No	133 (46.2)	155 (53.8)	

*Note:* Data are presented as mean ± SD for continuous variables. bold values indicate statically significant. The *p* value for a quantitative variable was calculated from the Mann–Whitney test. Qualitative variables *N* (%) obtained from the *χ* ^2^ analysis.

Abbreviations: BMI, body mass index; FFM, fat‐free mass; MAR, mean adequacy ratio; MD, Mediterranean diet; WC, wrist circumference.

**Table 2:** Dietary intake across case and control groups.

Variables	Case group (*N* = 174)	Control group (*N* = 171)	*p* value
	Mean ± SD	Mean ± SD	
Energy (kcal)	2402.62 ± 808.61	2148.80 ± 832.49	0.36
Carbohydrate (g)	307.34 ± 107.56	278.47 ± 103.56	0.05
Fat (g)	109.50 ± 48.92	94.60 ± 59.49	0.97
Protein (g)	88.42 ± 32.83	80.87 ± 34.67	0.90
Fiber (g)	28.73 ± 13.34	25.69 ± 12.55	0.92

*Note:* Data are presented as mean ± SD.

### 3.1 Associations Between MAR and MS

There was no significant difference in MAR between MS patients and the control group (Table 1). In addition, a significant difference was observed in age across the MAR tertiles, while no differences were observed in other variables (Table 3). Furthermore, total energy and macronutrient intake were significantly higher in the second or third tertile of MAR in comparison to the first (Supporting Information S1: Table S1). As was shown in Table 3, in the crude model, no significant association was detected between MAR and MS, while multivariate analysis revealed that higher MAR was significantly associated with lower odds of MS (*p* trend = 0.002). In addition, the second tertile of MAR and the third tertile of MAR were significantly associated with 50% and 75% lower odds of MS, respectively (Table 4).

**Table 3:** Characteristics of participants across tertiles of the scores.

Characteristics	MAR (*N* = 345)				MD (*N* = 345)			
Continuous variables	T1 (*N* = 115) (= < 1.14)	T2 (*N* = 115) (1.15–1.19)	T3 (*N* = 115) (> =1.20)	*p* value	T1 (*N* = 92) (score = < 3)	T2 (*N* = 153) (score 4–5)	T3 (*N* = 100) (score 6–8)	*p* value
	Mean ± SD	Mean ± SD	Mean ± SD		Mean ± SD	Mean ± SD	Mean ± SD	
Age (year)	33.95 ± 8.31	31.14 ± 9.48	32.23 ± 7.94	0.006	31.52 ± 8.56	32.49 ± 8.48	33.22 ± 9.02	0.51
Weight (kg)	66.20 ± 14.18	65.62 ± 14.01	66.01 ± 15.23	0.91	65.64 ± 15.51	65.49 ± 14.94	66.92 ± 12.64	0.54
Height (cm)	160.96 ± 8.28	163.71 ± 8.60	162.41 ± 8.74	0.05	162 ± 69 ± 9.93	161.09 ± 7.70	164.00 ± 8.34	**0.01**
BMI (kg/m^2^)	25.61 ± 5.41	24.67 ± 5.42	24.95 ± 5.15	0.44	24.75 ± 5.12	25.18 ± 5.19	25.22 ± 5.76	0.82
WC (cm)	84.39 ± 12.49	82.24 ± 12.06	82.48 ± 13.78	0.23	83.53 ± 13.13	82.05 ± 12.85	84.14 ± 12.42	0.40
Fat (%)	28.89 ± 8.31	26.75 ± 8.43	27.64 ± 8.50	0.19	26.99 ± 8.93	28.57 ± 8.01	27.23 ± 8.58	0.23
FFM (%)	44.22 ± 8.49	45.74 ± 9.60	44.80 ± 8.80	0.79	44.85 ± 9.95	44.20 ± 8.49	46.18 ± 8.70	0.34

Categorical variables	*N* (%)	*N* (%)	*N* (%)	*p* value	*N* (%)	*N* (%)	*N* (%)	*p* value
Gender								
Female	94 (34.3%)	86 (31.4%)	94 (34.3%)	0.32	68 (24.8)	132 (48.2)	74 (27)	**0.01**
Male	21 (29.6%)	29 (40.8%)	21 (29.6%)		24 (33.8)	21 (29.6)	26 (36.6)	
Smoking
Yes	21 (37.5)	13 (23.2)	22 (39.3)	0.20	18 (32.1)	23 (41.1)	15 (26.8)	0.60
No	94 (32.6)	102 (35.5)	92 (31.9)		74 (25.7)	130 (45.1)	84 (29.2)	

*Note:* Bold values indicate statically significant.

**Table 4:** Association between the tertiles of MAR and MS.

Variables	T2		T3		
	OR (95% CI)	*p* value	OR (95% CI)	*p* value	*p* trend
Crude	0.87 (0.51, 1.45)	0.59	1.03 (0.61, 1.73)	0.89	0.89
Adjust	0.50 (0.27, 0.95)	**0.03**	0.25 (0.10, 0.62)	**0.002**	**0.002**

*Note:* Reference group: first tertiles. bold values indicate statically significant. All data are presented as OR and 95% Cl by binary logistic regression. Adjustment model: based on age, smoking, energy intake, and sex.

### 3.2 Associations Between MD and MS

**Figure 2:**
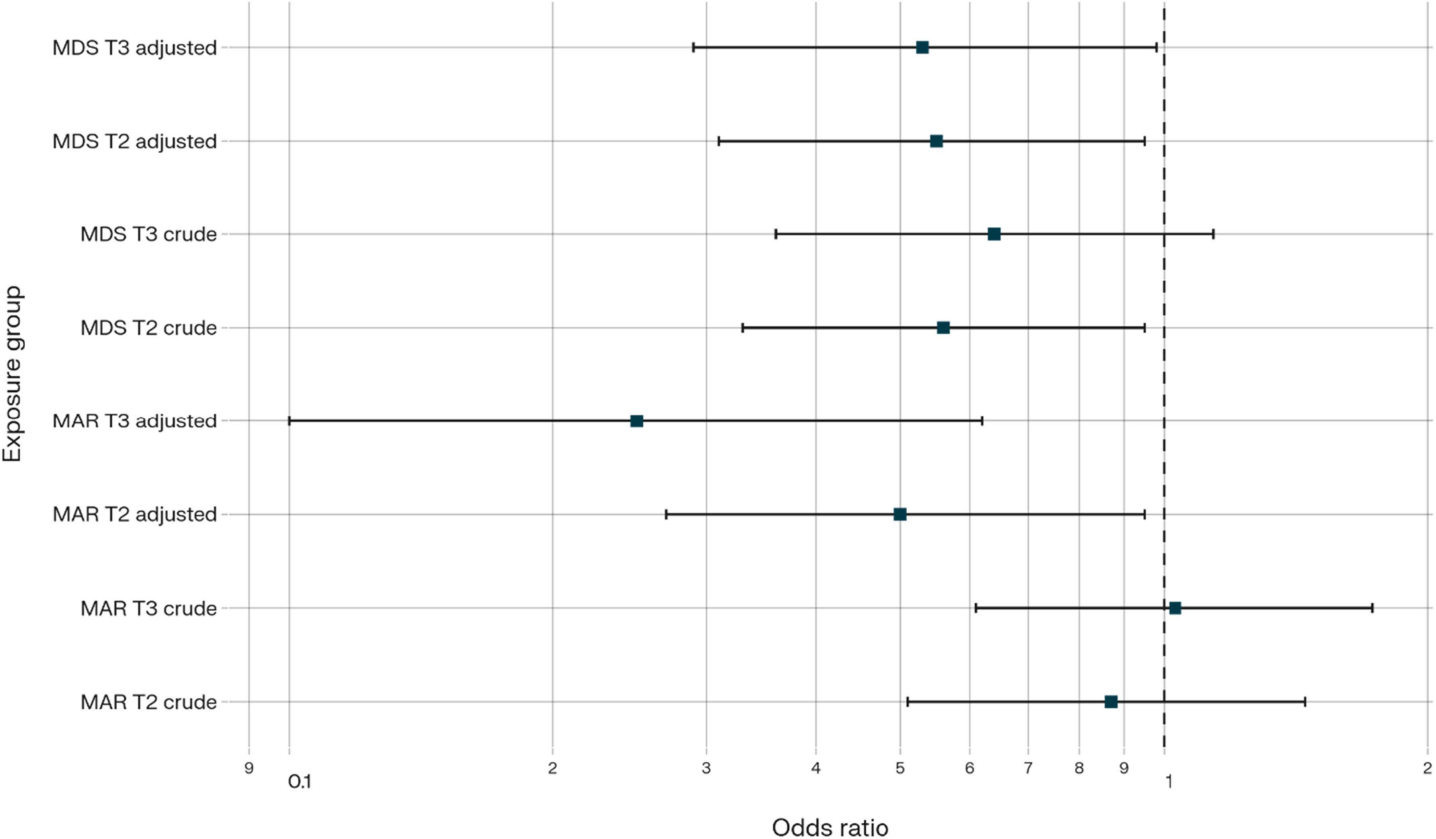
Forest plot. Squares represent point estimates (OR); horizontal lines represent 95% CIs. The vertical dashed line indicates OR = 1.0 (no association). Logarithmic scale on the x‐axis.

No significant difference in MD was observed between individuals with MS and the control group (Table 1). A significant difference was detected in the prevalence of genders across the tertiles of MD. Also, height was significantly higher in the highest tertile of MD than lowest. However, no significant difference was detected in other variables between MD tertiles (Table 3). In addition, energy intake as well as each macronutrient consumption were significantly higher in the highest tertile of MD tertiles compared to the first (Supporting Information S1: Table S2). Binary logistic showed that adherence to the MD as the second tertile, in contrast to the third, was significantly associated with 44% lower odds of MS. Furthermore, multivariable analysis revealed that adherence to higher MD was significantly associated with lower odds of MS. In addition, it was shown that adherence to the MD as second and third tertiles was significantly related to the 45% and 47% lower odds of MS, respectively (Table 5, Figure 2).

**Table 5:** Association between the tertiles of MD and MS.

Variables	T2		T3		
	OR (95% CI)	*p* value	OR (95% CI)	*p* value	*p* trend
Crude	0.56 (0.33, 0.95)	0.03	0.64 (0.36, 1.14)	0.13	0.14
Adjust	0.55 (0.31, 0.95)	0.03	0.53 (0.29, 0.98)	0.04	0.04

*Note:* Reference group: first tertiles. All data are presented as OR and 95% Cl by binary logistic regression. Adjustment model: based on age, smoking, energy intake, and sex.

## 4 Discussion

The present case‐control study provides compelling evidence that higher adherence to the MeDi, as measured by the MDS, and better micronutrient adequacy, as assessed by the MAR, are associated with a reduced risk of MS. Specifically, individuals in the highest tertiles of MDS and MAR exhibited 47% and 75% lower odds of having MS, respectively, after adjusting for potential confounders such as age, sex, smoking status, and energy intake. These findings underscore the potential of diet as a modifiable risk factor in MS prevention, offering valuable insights for public health strategies.

Our results are consistent with prior research highlighting the beneficial effects of the MeDi on MS outcomes. For instance, a pilot randomized controlled trial, complemented by two cross‐sectional studies, indicated that greater adherence to the MeDi was associated with diminished disease severity, reduced disability, and lower levels of fatigue and depression among individuals with MS [20, 21, 22]. Similarly, a study on pediatric‐onset MS found that higher MDS and increased intake of specific nutrients were linked to a lower probability of developing the disease [23]. A recent prospective study suggested that dietary patterns resembling the MeDi may play a protective role against MS development [10]. These consistent findings across different study designs strengthen the evidence base for the role of diet in MS, emphasizing the need for dietary interventions as part of MS management.

The biological plausibility of these associations can be attributed to the anti‐inflammatory and antioxidant properties of the Mediterranean diet. Its high content of fruits, vegetables, and olive oil provides vitamins C, A, E, folate, and polyphenols, which combat oxidative stress and inflammation, protecting neural tissues and supporting immune function, with studies linking higher intakes to reduced MS progression [24, 25, 26, 27]. Whole grains, legumes, and nuts supply dietary fiber, magnesium, zinc, and B vitamins (niacin, riboflavin, thiamin), promoting a healthy gut microbiome that modulates immune responses and reduces neuroinflammation, with fiber intake associated with lower MS odds [28, 29, 30, 31]. Omega‐3 fatty acids from fish reduce pro‐inflammatory cytokines (e.g., TNF‐α, IL‐6) and enhance anti‐inflammatory mediators, while olive oil's monounsaturated fats and polyphenols further mitigate inflammation [32, 33, 34, 35]. Additionally, the diet's low consumption of ultra‐processed foods, saturated fats, and sodium minimizes gut dysbiosis and immune dysregulation, potentially delaying MS onset [36]. Collectively, these components ensure comprehensive micronutrient adequacy, supporting the protective role of the MeDi in MS, as evidenced by the associations between MAR nutrients and reduced disease risk [37].

Notably, this study is innovative in simultaneously evaluating both MDS and MAR, addressing a gap in the literature where dietary patterns and nutrient adequacy are often examined in isolation. By integrating these metrics, our findings highlight the synergistic effects of overall dietary quality and specific nutrient intake on MS risk. However, several limitations must be acknowledged. As a case‐control study, our design is susceptible to recall bias, particularly in the assessment of dietary intake, which relied on self‐reported data. While we adjusted for key confounders, residual confounding cannot be entirely ruled out. Furthermore, the study was conducted in Iran, where cultural dietary practices may differ from other regions, potentially limiting the generalizability of our findings. For example, while the Iranian diet shares similarities with the MeDi in terms of vegetable and legume consumption, it may differ in fish and olive oil intake, which are central to the traditional Mediterranean pattern [38, 39]. Future research should aim to replicate these results in diverse populations to confirm the universality of the observed associations. Additionally, the cross‐sectional nature of our study precludes the establishment of causality. Longitudinal studies are essential to determine whether adherence to the MeDi and adequate micronutrient intake precede MS onset. Intervention trials could further elucidate the potential of dietary modifications as a preventive strategy for MS, particularly in high‐risk populations. Such studies could also explore the impact of region‐specific dietary adaptations on MS risk, ensuring that recommendations are culturally relevant and feasible [40, 41].

## 5 Conclusion

In conclusion, our findings suggest that higher MDS and MAR are associated with a reduced risk of MS, providing further support for the role of diet in MS prevention. Public health initiatives should consider promoting Mediterranean dietary patterns and ensuring adequate micronutrient intake, particularly in populations at higher risk for MS. Integrating dietary counseling into MS care, tailored to optimize micronutrient adequacy, could complement existing treatment strategies. Future research should focus on longitudinal and interventional studies to establish causality and to explore the mechanisms underlying these associations, paving the way for evidence‐based dietary guidelines in MS prevention and management.

## Author Contributions

**Maryam Ahmadi‐Khorram:** conceptualization, data curation, investigation, project administration, supervision, writing – review and editing, writing – original draft, visualization. **Alireza Hatami:** conceptualization, data curation, investigation, project administration, supervision, writing – original draft, writing – review and editing. **Mostafa Shahraki Jazinaki:** writing – original draft, writing – review and editing. **Yasaman Aali:** writing – original draft, writing – review and editing, software, formal analysis. **Maedeh Nojoumi:** writing – review and editing. **Fatemeh Keykhaei:** writing – review and editing, writing – original draft, data curation, investigation. **Ali Jafarzadeh Esfehani:** writing – original draft, writing – review and editing, methodology. **Mohsen Nematy:** writing – review and editing, supervision, funding acquisition, resources, validation.

## Ethics Statement

The research received approval from the Research Committee of the Mashhad University of Medical Sciences, Iran. The study adheres to the principles outlined in the Declaration of Helsinki. All participants provided written informed consent before participating in this study. Moreover, it was approved by the Ethics Committee of the Research Vice‐Chancellor at Mashhad University of Medical Sciences (IR.MUMS.REC.1393.182), and all personal information about participants will be kept secure in a database.

## Consent

The authors have nothing to report.

## Conflicts of Interest

The authors declare no conflicts of interest.

## Transparency Statement

The corresponding author, Dr. Mohsen Nematy, affirms that this manuscript is an honest, accurate, and transparent account of the study being reported; that no important aspects of the study have been omitted; and that any discrepancies from the study as planned (and, if relevant, registered) have been explained.

## Supporting information


Supporting File

## Data Availability

Data described in the manuscript, will be made available upon written request to the corresponding author.
