# Supplementary material for: Mediterranean Diet and Micronutrient Adequacy in Relation to Relapsing‐Remitting Multiple Sclerosis Risk: A Case‐Control Study
Source: Health Sci Rep. 2026 Aug 30;9(9):e73139. doi: 10.1002/hsr2.73139 (PMC13527407; doi:10.1002/hsr2.73139)
Supplement: Supplementary file 1 [file HSR2-9-e73139-s001.docx]

**Running Title:** Diet Reduces MS Risk

**Mediterranean Diet and Micronutrient Adequacy Reduce Multiple Sclerosis Risk**

*Maryam Ahmadi-Khorram^1,2†^, Alireza Hatami^1,2†^, Mostafa Shahraki-Jazinaki^1,2^,* *Yasaman Aali^1,2^, Maede Nojoumi^1,2^, Fatemeh Keykhaei^1,2^, Mohsen Nematy^1,3*^*

**^1^** Department of Nutrition, Faculty of Medicine, Mashhad University of Medical Sciences, Mashhad, Iran.

**^2^** Student Research Committee, Mashhad University of Medical Sciences, Mashhad, Iran.

**^3^** Metabolic Syndrome Research Center, Mashhad University of Medical Sciences, Mashhad, Iran

**Emails:** [hatami.alireza031@gmail.com](mailto:hatami.alireza031@gmail.com), [maryamahmadikhorram@gmail.com](mailto:maryamahmadikhorram@gmail.com), [NematyM@mums.ac.ir](mailto:NematyM@mums.ac.ir)

^†^ **First authorship**

**^*^ Corresponding Author**

***Dr Mohsen Nematy (MD, PGDip., PhD)***

Professor in Clinical Nutrition

Department of Nutrition, School of Medicine

Chairperson: Journal of Nutrition, Fasting, and Health, <https://jnfh.mums.ac.ir/>

Metabolic Syndrome Research Center, Mashhad University of Medical Sciences (MUMS)

Postcode 91779-48564, Mashad, Iran

Tel: +98 (51) 38002103 (direct), 38002107 & 38827033 (secretary)

E-mail: [NematyM@mums.ac.ir](mailto:NematyM@mums.ac.ir)

Orcid iD**:** <https://orcid.org/0000-0003-3202-2709>

**Supplementary materials**

| **Table S1.** Dietary intake across tertiles of MAR. | | | | |
| --- | --- | --- | --- | --- |
| **Variables** | T1 (N=115) | T2 (N=115) | T3 (N=115) | P-value ^*^ |
|  | Mean±SD | Mean±SD | Mean±SD |  |
| Energy (Kcal) | 1541.57 ± 457.75 | 2226.22 ± 489.06 ^a^ | 3062.64 ± 674.49 ^b^ | **<0.001** |
| Carbohydrate (g) | 206.97 ± 65.97 | 283.69 ± 72.00 | 388.43 ± 88.91 | **<0.001** |
| Fat (g) | 62.23 ± 26.81 | 102.18 ± 43.90 | 140.93 ± 58.18 | **<0.001** |
| Protein (g) | 53.94 ± 17.06 | 84.03 ± 22.21 | 116.07 ± 27.18 | **<0.001** |
| Fiber (g) | 17.00 ± 6.33 | 25.84 ± 9.40 | 38.83 ± 11.86 | **<0.001** |
| Data are presented as mean ±SD.  *) P-value obtained by performing the Kruskal-Wallis Test  Significant items with P < 0.05 are bolded.  a: significant compared to tertile 1  b: significant compared to tertile 1 | | | | |

| **Table S2.** Dietary intake across tertiles of MD. | | | | |
| --- | --- | --- | --- | --- |
| **Variables** | T1 (N=92) | T2 (N=153) | T3 (N=100) | P-value ^*^ |
|  | Mean±SD | Mean±SD | Mean±SD |  |
| Energy (Kcal) | 2148.11±806.21 | 2205.13±874.45 | 2504.89±735.83 | **0.001** |
| Carbohydrate (g) | 271.69±99.95 | 282.80±105.96 | 328.32±105.33 | **0.005** |
| Fat (g) | 100.47±58.00 | 100.12 ± 60.80 | 106.69 ± 40.67 | **<0.001** |
| Protein (g) | 78.145±33.22 | 83.65±35.93 | 92.25±30.03 | **0.01** |
| Fiber (g) | 21.18±9.15 | 26.16±11.42 | 34.41±14.98 | **0.01** |
| Data are presented as mean ±SD.  *) P-value obtained by performing the Kruskal-Wallis Test  Significant items with P < 0.05 are bolded.  a: significant compared to tertile 1  b: significant compared to tertile 1 | | | | |
